# Enhancing clomipramine antidepressive effect using nanocomplexes via different routes of administration: A comparative study

**DOI:** 10.22038/ijbms.2025.88730.19176

**Published:** 2026

**Authors:** Abeer Salama, Rabab Kamel, Nourina N. Ghoneim, Shereen S. Elshaer

**Affiliations:** 1 Pharmacology Department, National Research Centre, El-Bohooth Street (P.O. 12622), Cairo, Egypt; 2 Pharmaceutical Technology Department, National Research Centre, El-Bohooth Street, Cairo, Egypt; 3 Egyptian Drug Authority, Cairo, Egypt; 4 Department of Biochemistry, Faculty of Pharmacy, Heliopolis University, Cairo, Egypt; 5 Department of Biochemistry and Molecular Biology, Faculty of Pharmacy (Girls), Al-Azhar University, Cairo, Egypt

**Keywords:** Chitosanmm, Ciprofloxacin, Clomipramine, Depression, Gum Arabic, GAPA/EAAT2, Nanocomplex

## Abstract

**Objective(s):**

Enhancing clomipramine anti-depressive effect using a nanoformulation and investigating its effect via different routes of administration (oral and intranasal) in a depression rat model. Polyelectrolytes nanocomplexes (NC) were prepared by an all-aqueous technique and were composed of different ratios of chitosan (CS) and gum arabic (GA).

**Materials and Methods:**

Ciprofloxacin (CPX) was administered orally to adult male Wistar albino rats at a dose of 50 mg/kg for 14 days to induce depression. Clomipramine HCl solution and the drug-loaded nano complexes (NC) were administrated for 14 days via the oral route (50 mg/kg) and intranasal route (500 µg/kg).

**Results:**

All the prepared drug-loaded nano-complexes (NC) were uniformly distributed (PDI ˂0.2), NC1 (composed of CS:GA 1:1) attained the smallest particle size (200.30 ± 26.07nm) and the most sustained release profile (Mean Release Time= 96.02± 8.36 min.) and has a spherical outline as detected by transmission electron microscope. Treatment with clomipramine-loaded NC via oral and intranasal routes elevated swimming time, serotonin (5-HT), excitatory amino acid transporter 2 (EAAT2) and Gamma-aminobutyric acid (GABA) brain contents, decreased brain content of malondialdehyde (MDA) and nitric oxide (NO), and ameliorated nuclear pyknosis and degeneration of neurons compared to CPX and clomipramine solution. Clomipramine-loaded NC via intranasal routes returned the brain content of 5-HT and EAAT 2 to its normal level and has effect superior than oral route.

**Conclusion:**

Clomipramine-loaded NC administered via intranasal route showed an enhanced effect and a higher antidepressant activity than the traditional oral route through alleviating CPX neurological toxicity.

## Introduction

Depression is a frequent mental condition that impairs function and leads to early death. It is clinically defined by a depressed mood, anhedonia (loss of interest or pleasure), and recurring feelings of guilt, interrupted sleep and eating, lack of energy, and difficulty concentrating. These symptoms might develop into chronic or recurring conditions that make it impossible for the affected person to handle their everyday obligations (1). One of the most significant issues in Arab nations is the elevation of depression prevalence. Researchers conducted a study to determine the rate of depression among Egyptian university students throughout the COVID-19 pandemic, which showed one-third had moderate to severe depression (2). More than 22% of Egyptian house officers experienced depression during the COVID-19 pandemic (3). Being female, unemployed, and divorced are just a few social and demographic factors that have been linked to depression and suicide (4).

Oxidative stress induces lipid peroxidation, which in turn impairs neuronal communication, damages cell membranes, and causes neuronal injury, particularly in the hippocampus, which is responsible for mood regulation and is affected in depression. NO regulates neuroplasticity, neurogenesis, and neurotransmitters involved in depression, including serotonin, dopamine, and glutamate (5).

Ciprofloxacin (CPX), an antibacterial drug, is frequently prescribed in clinical settings. In the literature, only a few instances of psychosis caused by ciprofloxacin have been documented; in addition, headache, vertigo, and sleeplessness are the most frequent adverse reactions (6). The predicted overall incidence of adverse consequences is 9.3% (7). A tricyclic antidepressant (TCA), such as clomipramine, acts on about five separate neurotransmitter pathways to produce its effects. The drug is thought to alleviate depression primarily by elevating the levels of norepinephrine and serotonin available in the synapses. It also works by blocking key postsynaptic receptors, specifically the H1 histamine receptor, the muscarinic receptor, and both alpha-1 and alpha-2 cholinergic receptors (8). 

Nanomedicine has been the focus of many researchers for the management of many diseases (9). Many techniques can be used to prepare nanoparticles; however, most require organic solvents and heat, which pose significant disadvantages. Another method based on self-assembly via interpenetrating polyelectrolyte complexation has proved to be a successful approach for the preparation of nanocomplexes, offering many advantages, such as being simple, cost-effective, all-aqueous, and safe, as well as a convenient method for the sustained release of water-soluble drugs (10-12). The obtained nano complexes (NC) are stable and a homogenous colloidal dispersion (13). These materials are created by mixing oppositely charged polysaccharides, such as positively charged chitosan (CS) and negatively charged Gum Arabic (GA). The resulting water-insoluble copolymeric network forms in aqueous solution, allowing for controlled drug release (11, 14). 

Clomipramine is an effective antidepressant with potent serotonin reuptake inhibition and has established efficacy. Its side effect profile with oral administration is a major limitation, which includes anticholinergic symptoms such as dry mouth, blurred vision, weight gain, constipation, and various cardiovascular problems. The rationale for choosing clomipramine and the intranasal route is likely rooted in a desire to address these very issues and explore a novel therapeutic approach (15). 

For more than three decades, the nasal cavity has been extensively studied as a potential alternative route of administration for medications with systemic effects, bypassing oral or parenteral routes. It is regarded as a quick route because of its highly vascularized mucosa, which enhances bioavailability, particularly for larger drug molecules, and enables targeted drug delivery to the brain (16). For this reason, we aimed to prepare a nanoparticulate system to increase the antidepressive effects of clomipramine. 

## Materials and Methods

### Animals

The study used adult male Wistar Albino rats (120−140 g), which were sourced from the National Research Centre (NRC, Dokki, Giza, Egypt). All animal experiments adhered strictly to the ethical standards set by the NRC Medical Committee and the ARRIVE guidelines. Furthermore, the procedures conformed to the National Institutes of Health Guide for the Care and Use of Laboratory Animals (NIH publication no. 8023, revised 1978).

### Materials

Chitosan (medium molecular weight) was purchased from Sigma-Aldrich (Germany). Gum Arabic was of a commercial grade. Clomipramine HCl was obtained from the Novartis company, CPX was obtained from MEMCO (Memphis Co. for Pharm. & Chem. Ind.) in Cairo, Egypt. EAAT 2 and GABA kits were purchased from NOVA kits (Beijing, China), MDA and NO kits were purchased from Biodiagnostic kits, Egypt.

### Preparation of the nanocomplexes

**Figure 1:**
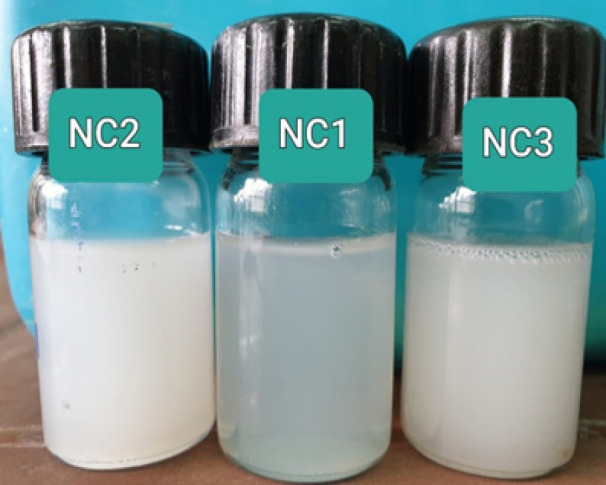
Images of the prepared nanocomplexes (NCs)

 method based on ionic cross-linking was followed with some modifications (12). Briefly, CS and GA were dissolved in distilled water separately (1% w/v). The drug (10 mg/ml) was dissolved in the CS aqueous dispersion, which was then added dropwise to the GA aqueous dispersion under continuous magnetic stirring. Addition was performed at a constant rate of 1 ml/min at room temperature using a magnetic stirrer (Heidolph, Germany) (17). The composition of the prepared nanocomplexes is listed in Table 1. The pH values of the preparations were adjusted to around 5.5 to facilitate the complexation and tightening of the formed nano-composite network (11). At this pH, the cationic CS is ionized (pKa = 6.3) (18). Also, the anionic GA is negatively charged at this pH (pKa=2.2) as the carboxylic groups of the glucuronic acid residue are deprotonated (19, 20), which favors the inter-polymeric bonding. The images of the prepared nanocomplexes are shown in Figure 1.

### Drug entrapment

To determine the entrapped drug, a 100 mg sample of the preparation was dissolved in 10 ml of distilled water. This was achieved by sonication at 70 °C for two hours to ensure the drug was fully extracted. The resulting solution was then filtered through a 0.45 μm Millipore filter. Finally, the drug content was assayed by UV-spectrophotometry (Shimadzu UV Spectrophotometer) at 265 nm after appropriate dilution. The % drug entrapped was calculated as follows: 

(Actual drug amount/ Theoretical drug amount) × 100 

### Particle size (PS), size distribution (PDI), and Zeta potential (Z) of the nanocomplexes

The mean particle size and size distribution of the prepared formulae were measured using the Malvern Zetasizer Nano ZS (Malvern Instruments Ltd., Malvern, UK). Before measurement, samples were diluted with distilled water (1:10).

### In-vitro release study

Drug release was performed by the dialysis method. The nanocomplexes (equivalent to 5 mg of drug) were placed in well-tightened dialysis bags immersed in 100 ml of phosphate buffer, pH 6.8 (21, 22). Shaking was done at 100 rpm, and the temperature was maintained at 37 °C. During the experiment, 2 ml samples were collected at predetermined intervals and replaced with an equal volume of fresh release medium. The drug concentration in each sample was measured at 265 nm using a Shimadzu UV spectrophotometer (2401/ PC, Japan). Release profiles were then plotted to determine the mean release time (MRT) using the provided equation:

$\mathrm{MRT}=(\sum t_{j}∆Q_{j})/\sum ∆Q_{j}$

Where *j *is the sample number, *tj *is the time at the midpoint between tj and tj–1, and ΔQj is the additional amount of drug dissolved in the period *tj *and *tj*–1. The MRT is a parameter to quantify the rate of drug release. A higher MRT indicates a slower release rate (23). 

### Transmission electron microscope (TEM)

The morphology of the selected nano-complex was analyzed using transmission electron microscopy (TEM) (JEM-1230, JEOL, Japan). The sample preparation involved diluting it with distilled water, placing the diluted sample onto carbon film-coated copper grids, and subsequently staining it with a 2% (w/w) phosphotungstic acid solution prior to examination.

### FTIR spectroscopy study

The drug, the selected nanocomplex, and the formula ingredients were mixed separately in a 100:1 ratio with IR-grade KBr, and corresponding discs were prepared using a hydraulic press. The discs were scanned over a wave-number range of 4000–400 cm^−1^ using a Shimadzu IR/FTIR spectrophotometer (Japan).

### In vivo experimental design

Depression was induced by the oral administration of CPX at a daily dosage of 50 mg/kg over a period of 14 days (24). After 2 weeks of acclimatization, 60 male Wistar albino rats were randomly divided into six groups (10 rats each): Group I: Rats were administered normal saline (5 ml/kg, orally, once a day) for 14 days and served as the control group. Group II: Rats received CPX at 50 mg/kg once daily for 2 weeks. Group III: Rats were administered orally CPX plus oral administration of clomipramine drug solution (50 mg/kg) over two weeks. Group IV: Rats administered orally CPX plus oral administration of clomipramine drug-loaded NC (50 mg/kg) over two weeks. Group V: Rats administered orally CPX plus intranasal administration of clomipramine drug solution (500 µg/kg; 1/10 of oral dose) (25) over two weeks.. Group VI: Rats administered orally CPX plus intranasal administration of clomipramine drug-loaded NC (500 µg/kg) over two weeks (Table 1).

### Forced swimming test (FST)

On day 15, the forced swim test (FST), a behavioral test, was conducted to test the antidepressant activity of the clomipramine drug solution and drug-loaded NC administered via the oral and intranasal route [28].

### Biochemical assays

After behavioral testing, the fasted rats were sacrificed under light ether anesthesia by cervical dislocation. Each brain was rapidly dissected, washed with ice-cooled physiological saline, and homogenized in 50 mM phosphate buffer to create a 20% (w/v) homogenate (26). The sample was centrifuged at 5,000×g for 5 min (Sigma, 2-16K). The resulting supernatant was quickly harvested and stored at −80 °C (27). The resultant supernatant was used to determine brain neurotransmitters 5-HT, GABA, and EAAT2 (28). Also, the levels of MDA and NO, oxidative stress markers, were assessed in brain homogenate (29, 30).

### Histopathology

Autopsy brain samples were taken and processed for histology. This involved fixation in 10% formol saline, subsequent dehydration, clearing in xylene, and paraffin embedding at 56 °C. Tissue blocks were sectioned at 4 μm and stained with Hematoxylin & Eosin for microscopic examination (31). 

### Statistical analysis

GraphPad Prism® software (version 5) was used to perform the statistical tests. All the values are presented as mean ± SD. Comparisons between different groups were carried out using one-way analysis of variance (ANOVA) followed by Fisher’s LSD test. The difference was considered significant when *P*<0.05. 

## Results

### Determination of drug entrapped

The percentage of drug entrapped was greater than 98% in all prepared nanocomplexes, indicating the well-incorporation of the drug within the interpenetrating copolymeric network.

### Particle size (PS), size distribution (PDI), and Zeta potential (Z) of NC

The PS values of the prepared NC are displayed in Table 1, ranging from 200.30 to 256.81 nm. The nano-complex (NC1) composed of an equi-proportion of the cationic and anionic polymer had the smallest particle size (*P*<0.05). On the other hand, the Z values of the prepared NCs are displayed in Table 1; all had high Z values (˃│30│ mV). While NC3 had a negative Z value. Also, the PDI values are listed in Table 1. Where all the NCs had low values (<0.2). 

### In vitro drug release study

**Figure 2:**
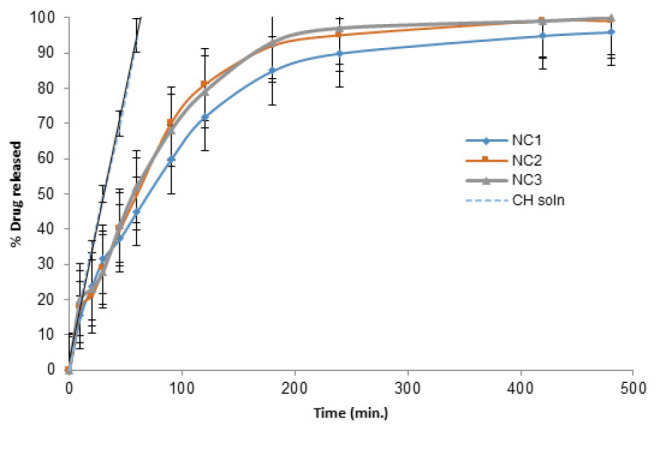
Release profiles of the prepared nanocomplexes compared to the CH solution

All the prepared NCs showed sustained-release profiles compared to the drug solution (Figure 2), and their MRT values (Table 2) were significantly higher (P<0.05), indicating slower drug release. Among the prepared nanocomplexes, NC1 attained the most sustained release profile. The value of the MRT of NC1 was significantly higher (*P*<0.05) compared to the other nanocomplexes. So, NC1 was selected for further investigation.

### Transmission electron microscope

**Figure 3:**
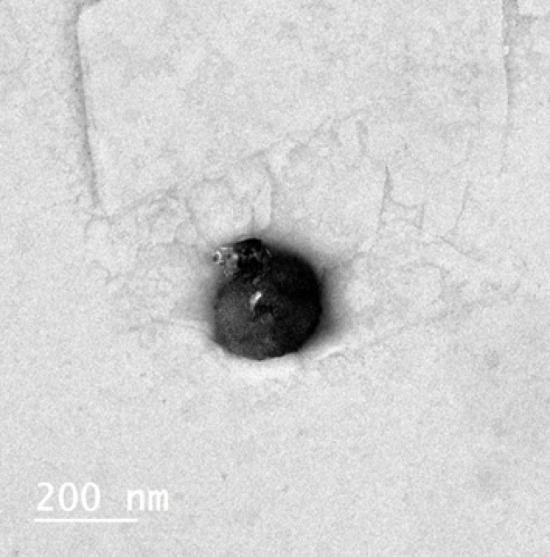
TEM photograph of the selected drug-loaded NC

Figure 3 displays the TEM photograph of the selected nanocomplex (NC1). Spherical black spots, having a diameter parallel to that recorded by the Zetasizer (Table 2), can be clearly seen.

### Forced swimming test

**Figure 4:**
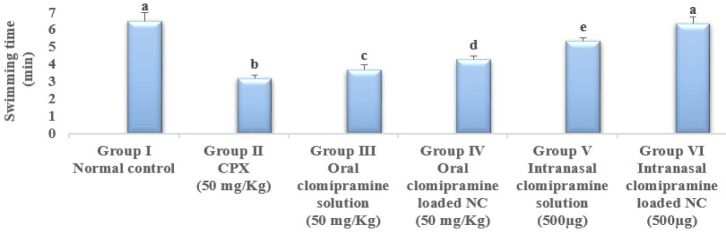
Effect of clomipramine treatment on the swimming time of rats

In this study, CPX reduced swimming time by 49% (3.19±0.21) compared with the normal control (6.52±0.50). The oral drug solution (50 mg/Kg) has prolonged swimming time by 15% (3.67±0.30). In comparison, the intranasal drug solution (50 mg/Kg) prolonged swimming time by 68% (5.36±0.20) compared with the CPX group (3.192±0.21), as shown in Figure 4. Treatment with clomipramine-loaded NC (500 µg) via oral route and intranasal routes prolonged the swimming time of rats by 34% (4.28±0.22) and 99% (6.36±0.4), respectively, as compared with the CPX group, where the intranasal drug-loaded NC returned the swimming time of rats to normal (Figure 4).

### Assessment of the brain content of the 5-HT neurotransmitter

**Figure 5:**
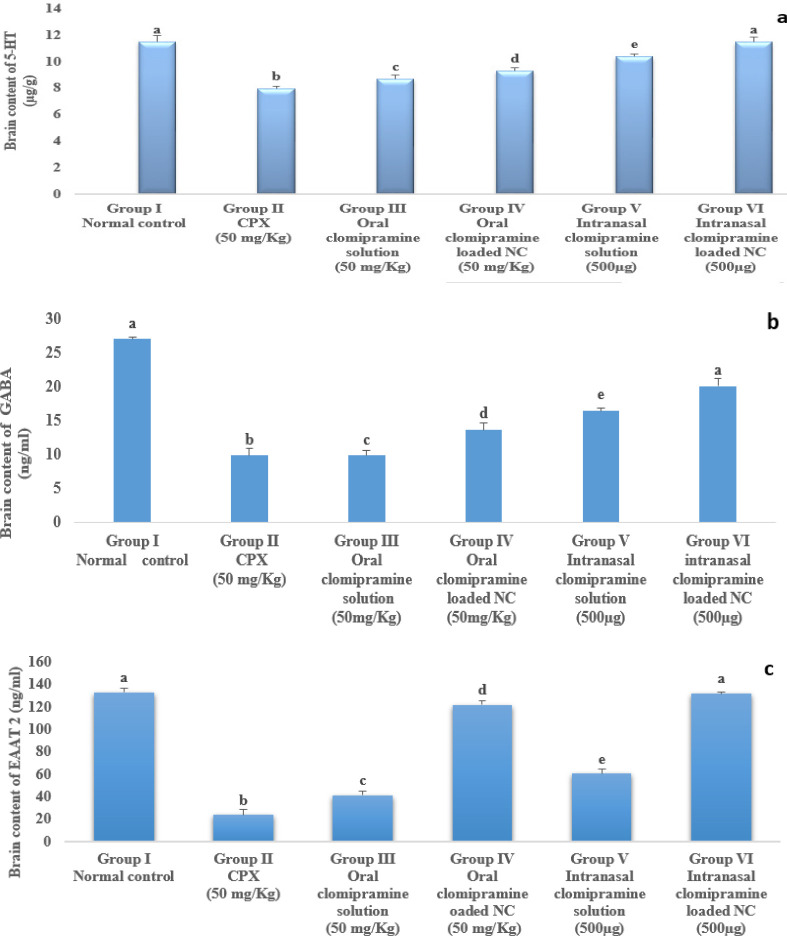
Effect of clomipramine treatment on brain content of a) 5-HT, b) GABA, c) EAAT2, d) MDA, and e) NO. Data were expressed as mean ±SD (n=10). The same letter means a non-significant difference, while a different letter means a significant difference at *P*<0.05.

The level of 5-HT was reduced in the CPX group by 31% (7.94±0.21 vs. 11.48±0.5) as compared with normal rats. The oral clomipramine solution (50 mg/Kg) has increased brain content of 5-HT by approximately 9% (8.67 ±0.3), while in the case of intranasal clomipramine solution (50 mg/Kg), brain content of 5-HT has increased by approximately 16% (9.28 ±0.22), after 14 days, as compared with the CPX group (7.94 ±0.21). Treatment with clomipramine-loaded NC (500µg) via oral and intranasal routes increased brain contents of 5-HT by approximately 30% (10.36 ±0.2) and 44% (11.64 ±0.4), respectively, as compared with the CPX group (7.94 ±0.21). The intranasal clomipramine-loaded NC returned the brain contents of 5-HT to its normal value (Figure 5a).

### Assessment of the brain content of Gamma-aminobutyric acid (GABA)

 In the current study, CPX decreased the brain content of GABA by 36% (9.8±1.06), as compared with the normal control group (26.95±0.3) (Figure 5b). The oral clomipramine solution (50 mg/Kg) has approximately no significant action on brain content of GABA (0.2%) (9.78±0.73). In contrast, in the case of intranasal clomipramine solution, there was an elevation of GABA brain content by 66% (16.36±0.46), as compared with the CPX group (9.80±1.06). Treatment with clomipramine-loaded NC (500 µg) via oral and intranasal routes, respectively, elevated brain content of GABA by 38% (13.56±1) and 104% (20.04±1.08), as compared with the CPX group (9.80±1.06) (Figure 5b).

### Assessment of brain content of excitatory amino acid transporter 2 (EAAT 2)

In the current study, CPX decreased the brain content of EAAT2 by 17.6% (23.33±4.71), as compared with the normal control group (132.67±4.18). Treatment with clomipramine solution (50 mg/kg) via oral and intranasal routes reveals that the oral solution elevates brain EAAT2 content by 75% (41.00±3.46). In contrast, the intranasal drug solution increased the brain content of EAAT2 by 158% (60.33±4.31) compared with the CPX group (23.33±4.71), as shown in Figure 5. Treatment with clomipramine-loaded NC (500 µg) via oral and intranasal routes, respectively, elevated brain content of EAAT 2 by 422% (121.80±3.41) and 464% (131.70±1.16), as compared with the CPX group (23.33±4.71). In addition, intranasal NC loading restored brain EAAT2 content to its normal level (Figure 5c).

### Assessment of CPX the brain content of MDA and NO

**Figure 6:**
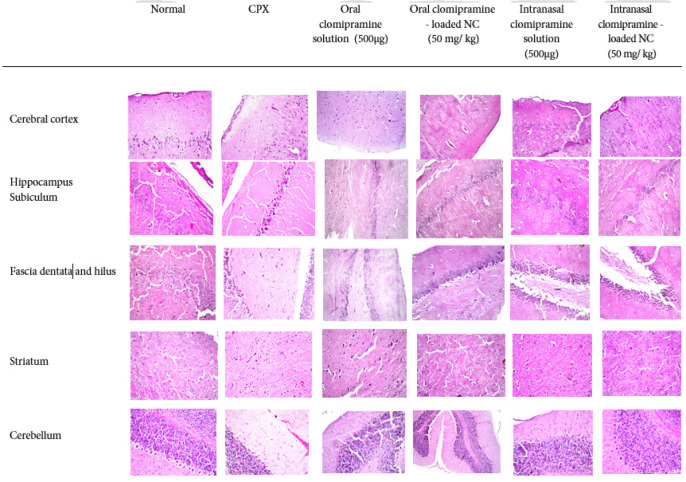
Photomicrograph of different sections of brain (a) A control rat, b) A rat treated with CPX, (C) A rat treated with oral administration of clomipramine drug solution, (d) A rat treated with oral administration of drug-loaded nano clomipramine, (e) A rat treated with intranasal administration of clomipramine drug solution, (f) A rat treated with intranasal administration of drug-loaded nano clomipramine.

In the current study, CPX elevated brain MDA and NO (a marker of lipid peroxidation) by 516% (10.17±0.05) and 52% (122.08±0.61), respectively, compared with the normal control (1.65±0.01) and (80.47±9.6), respectively. Treatment with clomipramine solution (50 mg/kg) via oral and intranasal routes decreased brain content of MDA by 95% (9.69±0.03) and 91% (9.29±0.07), Srespectively, compared to the CPX group (10.17±0.05), as shown in Figure 6a. Treatment with clomipramine-loaded NC (500 µg) via oral and intranasal routes, respectively, decreased brain content of MDA by 80% (8.23±0.07) and 76% (7.77±0.07), respectively, compared to the *VS* CPX group (10.17±0.05) (Figure 5d). Treatment with clomipramine solution (50 mg/kg) via oral and intranasal routes decreased brain NO content by 95% (116.33±0.04) and 89% (109.10±0.43), respectively, compared with the *VS* CPX group (122.08±0.61). Treatment with clomipramine-loaded NC (500 µg) via oral and intranasal routes decreased brain content of NO by 88% (109.10±0.43) and 72% (87.86±0.96), respectively, compared to the *VS* CPX group (122.08±0.61) (Figure 5e).

### Histological results

Control Rat (Healthy): Neurons were normal, showing no histopathological alteration in the cerebral cortex and subiculum area. CPX-Treated Rat (Disease Model): cerebral cortex showed severe damage, including nuclear pyknosis and diffuse neuronal degeneration, with focal inflammatory cell aggregation, also in the subiculum. Most of the neurons showed nuclear pyknosis and degeneration. Oral Clomipramine Solution: Provided little protection, with most neurons still exhibiting nuclear in the cerebral cortex, and showed some neurons that still appear dark and small, with pyknosis and degeneration in the subiculum area. Oral Nano-Clomipramine: Demonstrated a marked improvement in the damaged tissue, with no histopathological alteration noted in the cerebral cortex, and most of the neurons appear normal, with only a few that are still deeply stained. in subiculum area. Intranasal Clomipramine Solution: Showed a normal structure of the cerebral cortex and in the subiculum area. Intranasal Nano-Clomipramine: Also showed a quite normal structure of the cerebral cortex and the subiculum.

Control Rat (Healthy): Neurons were normal, showing no histopathological alteration in the fascia dentata, hilus area, striatum, cerebrum, and cerebellum. A rat with CPX-induced neurotoxicity showed that most neurons exhibited nuclear pyknosis, and degeneration was detected in some neurons in the fascia dentata, hilus area, and striatum, as well as focal hemorrhage in the fissure between the cerebrum and cerebellum. A rat treated with oral administration of clomipramine drug solution shows no histopathological alteration in the fascia dentata, hilus area, and shows nuclear pyknosis and degeneration in the striatum, and no histopathological alteration in the cerebellum. A rat treated with oral administration of drug-loaded nanoclomipramine shows nuclear pyknosis and degeneration in a few neurons in the fascia dentata and hilus areas, diffuse nuclear gliosis between intact neurons in the striatum, and no histopathological alteration in the cerebellum. A rat treated with intranasal administration of clomipramine drug solution showed nuclear pyknosis and degeneration in a few neurons in the fascia dentata and hilus areas, diffuse gliosis between intact neurons in the striatum, and no histopathological alteration in the cerebellum. A rat treated with intranasal administration of drug-loaded nano clomipramine shows no histopathological alteration of the fascia dentata and hilus, and a few gliosis between the intact neurons in the striatum, and no histopathological alteration in the cerebellum (Figure 6).

## Discussion

Depression is one of the most prevalent mood disorders with a high incidence of relapse and lifespan reduction (32). A TCA called clomipramine affects serotonergic neurotransmission. Although clomipramine has been used to treat a variety of mental illnesses both temporarily and permanently, it has proven to be very successful in treating obsessive-compulsive disorders (OCD). Clomipramine is a well-known TCA that suppresses NE & 5-HT reuptake. We chose to measure GABA and EAAT2 instead of NE, which is likely to provide a broader understanding of depression’s neurobiology for a long time. NE and 5-HT were the primary causes for understanding depression; however, more recent research has shown that depression is a complex disorder involving multiple interconnected neurotransmitter systems, including glutamate and GABA, which play a key role in the depression pathophysiology. So, we are focusing on novel therapeutic mechanisms by measuring GABA and EAAT2 levels; the researchers could be exploring whether clomipramine exerts its therapeutic effects by modulating these systems, providing new insights into the drug’s complete mechanism of action. Moreover, long-term administration of clomipramine may affect autophagy and, as a result, cellular homeostasis in the central nervous system (33). So, the present work studied the efficacy, greater targeting, and more significant effect of clomipramine-loaded NC via the intranasal route, which, in turn, improved the drug’s bioavailability.

All the prepared drug-loaded nanocomplexes were uniformly distributed (PDI ˂0.2), and NC1 (composed of CS:GA 1:1) attained the smallest particle size (200.30 ± 26.07nm). This may be due to the good fit of the anionic and cationic agents and to strong intermolecular interactions, which form a compact, well-arranged interpenetrating polymeric network. The increase in the proportion of either polymer leads to the agglomeration and accumulation of polymeric chains, thereby increasing the size of nanoparticles. Zeta potential values are known to indicate the stability of nanoparticles. Generally, charged particles having high zeta potential values are stable and have a low tendency for agglomeration because of the electrostatic repulsion between particles (34, 35). The current study demonstrated that all prepared NCs had high Z values (˃│30│ mV), indicating their stability. While NC3 had a negative Z value due to the higher proportion of the anionic polymers (GA). On the other hand, the low values of PDI (<0.2) of all the prepared NCs confirmed the uniformity and homogeneous size distribution of the prepared NCs. 

All the prepared NCs showed a sustained release pattern compared to the drug solution, and their MRT values were significantly higher (*P*<0.05), indicating slower drug release. The intense interaction between the COO- of GA and the NH3+ of CS, in addition to weaker Van der Waals forces and secondary hydrogen bonding, can allow the polymers to fit (36). Hence, this type of nanocomplexation can serve as a platform for sustained drug release. 

Among the prepared nanocomplexes, NC1 exhibited the most sustained release profile; this may be due to the tightness of the entangled, interpenetrating polymeric network formed by possible interactions between the oppositely charged polymers. The value of the MRT of NC1 was significantly higher (*P*<0.05) compared to the other nanocomplexes. This was selected for further investigation. 

The TEM photograph of the selected nanocomplex (NC1) shows clearly spherical black spots with diameters parallel to those recorded by the Zetasizer (Table 1). 

The forced swimming test is a behavioral model used to evaluate the potential serotonergic antidepressant effect by observing rats as they swim, struggle, and remain still (37). The current study revealed that after 14 days, CPX caused a decrease in swimming time and neurotransmitter signaling, such as 5-HT, which induced depression, in comparison to normal controls. CPX injection causes anxiety and depression by changing the amounts of 5-HT, dopamine, and noradrenaline in the brain (38). However, treatments with both oral and intranasal dosages of clomipramine solution increased swimming time and decreased 5-HT in the brain when compared with CPX rats. Moreover, in the current study, treatment with clomipramine-loaded NC via oral and intranasal routes showed a better and prolonged effect than oral and intranasal dosages of clomipramine solution, by elevating the swimming time of rats and the brain content of 5-HT. Clomipramine is primarily metabolized in the liver by CYP450 2D6 oxidation. It has a half-life of 17 to 28 hr. Then, CYP450 1A2 transforms clomipramine into the active, steady-state metabolite dimethyl clomipramine (39) that has more noradrenergic than serotonergic action. In cases with treatment-resistant OCD, specialists frequently combine clomipramine and the CYP450 1A2 inhibitor fluvoxamine to prevent its conversion to dimethyl clomipramine, which raises serotonergic activity (40). In the same time, intranasal delivery of antidepressants has several benefits over more conventional methods of delivery (oral, intravenous, intramuscular injection, etc.), including avoiding the first-pass effect, improving bioavailability, quick onset, small doses, fewer toxic and adverse effects on the body, and high patient compliance (39), reaching the central nervous system (CNS) and exhibit their antidepressant effects through the direct, non-invasive link between the nose and the brain. Drugs delivered to the CNS using nano vehicles exhibit unique properties such as continuous or delayed release, maintenance of the solubilized state, and improved penetration due to surface changes. All of these properties increase the effectiveness of delivery from the nose to the brain (40). Therefore, in the current study, clomipramine-loaded NC, administered at a dose of 500 µg /day intranasally, increased the drug’s bioavailability possibly by preventing the drug’s metabolism into dimethyl clomipramine by the liver’s first pass effect, and therefore, provided a more potent and long-lasting drug effect, which in turn can decrease any possible undesirable side effects by lowering its required dose. These facts explained the highest antidepressive effect of our formula, clomipramine-loaded NC, via the intranasal route.

Inhibitory GABA pathways also modulate stress response (41). It inhibits glutamatergic transmission and attenuates stress-induced NO release (42). EAAT2 is also a prospective target in mood disorders (43). The current work studied the effect of clomipramine on GABA and EAAT2 for the first time, treatment with clomipramine drug solution (50 mg/Kg) via oral and intranasal routes elevated the brain contents of GABA and EAAT2 after 14 days as compared with the CPX group, however, clomipramine drug loaded NC (500 µg) via oral and intranasal routes, respectively elevated brain contents of GABA and EAAT2, also, compared with CPX group. In addition, the intranasal drug-loaded NC has a superior effect than the clomipramine drug solution in elevating GABA and EAAT2 brain contents. These results can be explained by the nasal route offering better outcomes, with notable advantages including rapid and high systemic availability, avoidance of first-pass liver processing, and the potential to deliver drugs directly to the brain. So, intranasal clomipramine improved the effectiveness of clomipramine delivery and may have minimized adverse effects. 

Oxidative stress has a critical role in neuronal structural and functional alterations within the regions of the brain in neurodegenerative diseases like depression (44). In addition, chronic stress increases inducible nitric oxide synthase (iNOS) levels, thereby increasing NO production. Abnormal MDA levels have an essential role in depression (45, 46). In the current study, CPX elevated brain levels of MDA and NO compared with the normal control group, while clomipramine, administered orally and intranasally, decreased brain levels of MDA & NO compared with the CPX group. Also, clomipramine-loaded NC administered orally or intranasally decreased their brain contents. Conversely, it is plausible that several antidepressant classes (such as clomipramine) share a common mechanism: they boost anti-oxidant defenses and/or reduce pro-oxidant systems. This shared effect may be a key factor underlying the neuroprotective pharmacological impacts observed when these drugs are used clinically to treat stress-related disorders (39).

The current histopathological study also demonstrated that all clomipramine treatments, especially clomipramine-loaded NC (500 µg) via the intranasal route, nearly restored cortical tissue and reversed the severe neural injury, focal gliosis, degenerating neurons, apoptosis, and lysis caused by CPX administration. This effect of clomipramine may be attributed to the fact that clomipramine, which treats depression and OCD by antagonizing NE & 5-HT reuptake, has been shown to offer benefits in depression and reduce stress-induced deficits in rats at low chronic doses (47). Our findings confirm this, as clomipramine improved behavior in the FST, corrected levels of 5-HT, GABA, and EAAT neurotransmitters, and ameliorated oxidative stress, satisfying the criterion for predictive validity.

**Table 1:** Schematic of *in-vivo* experimental design of depression. CPX (Ciprofloxacin; 50 mg/kg) were administered orally for 2 weeks to induce depression. Rats were treated orally (50 mg/kg) and intranasally (500µg /kg) with clomipramine and NC (Nano complex).

	Groups		Days	Day 1- Day 14	15	16
ADULT MALE WISTAR ALBINO RATS (N=60)	Group 1 (Normal control)	(n=10)	Offering	Normal saline (5 ml/Kg), PO (once a day.	BEHAVIORAL TEST (FST)	BRAIN EXCISION AND PREPARATION
			Treatment			

	Group II	(n=10)	Offering	Oral administration of CPX (50 mg/kg) as a single daily dose		
			Treatment			

	Group III	(n=10)	Offering	Oral administration of CPX (50 mg/kg) as a single daily dose		
			Treatment	Oral administration of clomipramine solution (50 mg/kg(		

	Group IV	(n=10)	Offering	Oral administration of CPX (50 mg/kg) as a single daily dose		
			Treatment	Oral administration of clomipramine-loaded NC (50 mg/kg)		

	Group V	(n=10)	Offering	Oral administration of CPX (50 mg/kg) as a single daily dose		
			Treatment	Intranasal administration of clomipramine solution (500 µg)		

	Group VI	(n=10)	Offering	Oral administration of CPX (50 mg/ kg) as a single daily dose		
			Treatment	Intranasal administration of clomipramine-loaded NC (500 µg)		

CPX: Ciprofloxacin, NC: Nano complex, FT: Forced swim test

NC1, NC2 and NC3 where CS/GA ratio (w/w) = 1:1, 3:1 and 1:3, respectively

NC: Nano complex

**Table 2:** Composition, particle size (PS), Zeta potential (Z), and polydispersity index (PDI) of the drug-loaded nanocomplexes

Formulae	CS/GA ratio (w/w)	PS (nm)	Z (mV)	PDI	MRT (min)
NC1	1:1	200.30 ± 26.07	33.30 ± 5.36	0.166± 0.01	96.02± 8.36
NC2	3:1	247.62 ± 28.17	40.50 ± 4.57	0.167±0.01	77.34 ± 6.31
NC3	1:3	256.81 ± 29.96	-32.00 ± 7.59	0.186±0.01	75.00 ± 4.87
Drug solution					29.08 ± 3.36

CS: Chitosan; GA: Gum arabic

Data were expressed as mean ±SD (n=10). The same letter means a non-significant difference, while a different letter means a significant difference at *P*<0.05.

## Conclusion

The intranasal route can present a non-invasive way for bypassing the BBB and delivering therapeutic drugs directly to the CNS, providing a potential management of depression. The anti-depressant effect of the drug under study was enhanced by nanoencapsulation. Clomipramine HCl-loaded nanocomplexes could modify mood disorders induced by CPX in depressed rats through increasing swimming time and 5-HT, GABA, and EAAT2 brain contents, and scavenging reactive oxygen species.

## Data Availability

The datasets generated during the current study are available from the corresponding author upon request.

## Declaration

No AI tools or technologies were used to prepare this manuscript.
